# Reshaping healthcare delivery for elderly patients: the role of community paramedicine; a systematic review

**DOI:** 10.1186/s12913-020-06037-0

**Published:** 2021-01-06

**Authors:** Julia van Vuuren, Brodie Thomas, Gina Agarwal, Sean MacDermott, Leigh Kinsman, Peter O’Meara, Evelien Spelten

**Affiliations:** 1 Department of Community Health, Rural Health School, La Trobe University, Melbourne, Australia; 2 Department of Family Medicine, McMaster University, Hamilton, Canada; 3 Department of Health Research Methods, Evidence, and Impact, McMaster University, Hamilton, Canada; 4 University of Newcastle and Mid-North Coast Local Health District, Port Macquarie Base Hospital, Port Macquarie, Australia; 5 Department of Paramedicine, Monash University, Peninsula Campus, Melbourne, Australia

**Keywords:** Community paramedicine, Emergency medical technicians, Palliative care, Nursing home, Elderly, Health services for the aged, Terminal care

## Abstract

**Background:**

Healthcare systems are overloaded and changing. In response to growing demands on the healthcare systems, new models of healthcare delivery are emerging. Community paramedicine is a novel approach in which paramedics use their knowledge and skills beyond emergency health response to contribute to preventative and rehabilitative health. In our systematic review, we aimed to identify evidence of the community paramedicine role in care delivery for elderly patients, with an additional focus on palliative care, and the possible impact of this role on the wider healthcare system.

**Methods:**

A systematic review of peer-reviewed literature from MEDLINE, Embase, CINAHL, and Web of Sciences was undertaken to identify relevant full-text articles in English published until October 3, 2019. Additional inclusion criteria were studies focussing on extended care paramedics or community paramedics caring for elderly patients. Case studies were excluded. All papers were screened by at least two authors and underwent a quality assessment, using the Joanna Briggs Institute appraisal checklists for cross sectional, qualitative, cohort, and randomised controlled trial studies to assess the methodological quality of the articles. A process of narrative synthesis was used to summarise the data.

**Results:**

Ten studies, across 13 articles, provided clear evidence that Community Paramedic programs had a positive impact on the health of patients and on the wider healthcare system. The role of a Community Paramedic was often a combination of four aspects: assessment, referral, education and communication. Limited evidence was available on the involvement of Community Paramedics in palliative and end-of-life care and in care delivery in residential aged care facilities. Observed challenges were a lack of additional training, and the need for proper integration and understanding of their role in the healthcare system.

**Conclusions:**

The use of community paramedics in care delivery could be beneficial to both patients’ health and the wider healthcare system. They already play a promising role in improving the care of our elderly population. With consistent adherence to the training curriculum and effective integration within the wider healthcare system, community paramedics have the potential to take on specialised roles in residential aged care facilities and palliative and end-of-life care.

**Supplementary Information:**

The online version contains supplementary material available at 10.1186/s12913-020-06037-0.

## Background

In response to growing demands on the healthcare systems, new models of healthcare delivery are emerging [1–4]. The healthcare workforce, jurisdictional authorities and healthcare organisations are becoming more differentiated and embracing inter-professional collaboration and task substitution. This growing international trend in healthcare policy refers to a shift away from historical workforce hierarchies, and to allocating roles based on professional accomplishment [1, 4]. One of the areas currently undergoing rapid and significant change is delivery of care to elderly adults [2, 3].

It has been well established that the world’s population is ageing [5]. Elderly adults experience more illnesses which are often chronic conditions, such as cancer, or cardiovascular diseases [6]. They typically have multiple diseases (such as dementia, osteoporosis and arthritis) and the severity score of comorbidity increases with age [7, 8], requiring more complex and specialised care. Therefore, the already high demand on palliative care (PC), end-of-life care (EOL) (together: PEOL), and (specialised) aged care is likely to increase in the near future [8]. This increased demand will occur alongside a predicted healthcare workforce shortage. This is likely to prove particularly challenging for Residential Aged Care Facilities (RACFs), where the number of visiting physicians is forecast to decrease [2].

Elderly adults residing in RACFs are a complex and vulnerable population, with high levels of frailty, functional impairment, and comorbidities including cognitive impairment [9]. The number of people in residential aged care is increasing in Western countries and there are concerns about the care provided. Harrington et al. found that in their study standards and levels of care in most countries do not meet levels recommended by experts [10]. In Australia, aged care services admissions increased by 42% in 2017–18 [11] and the current Royal Commission into Aged Care has concluded that aged care is failing to meet basic community expectations and quality of care [12]. Some of the issues highlighted include patchy and fragmented palliative care and difficulties in recruiting and retaining adequately skilled staff [12].

Elderly adults, including RACF residents, comprise a considerable percentage of all emergency department presentations and hospital admissions [13–16]. Residents of RACFs experience higher hospital admission rates than community-dwelling elderly adults [17]. Yet, it is debatable whether emergency department visits and hospitalisation always lead to improved health outcomes for this vulnerable group. Emergency departments have been identified as potentially harmful environments for the elderly, especially those with dementia [3, 18]. Once admitted to hospital, elderly patients are more at risk of adverse events, such as delirium, functional decline, and readmission [15, 19].

Given the identified negative impact of hospitalisation on the elderly population, it has been suggested that ways to prevent ED presentation for non-urgent conditions, which might be better managed at place of residence, be explored [20, 21]. Several models of healthcare service delivery are emerging that challenge traditional professional boundaries [2]. As ED presentations of the elderly often coincide with Paramedic Service transport, this patient population has been associated with increased delays for Paramedic Services and the ED, leading to negative consequences for both patients and the Paramedic Service system [22, 23]. Community paramedicine (CPN) is a new approach aims to address this and other system shortfalls. In this extension of their scope of practice, paramedics use their knowledge and skills beyond emergency health response to introduce preventative and rehabilitative health. They are also involved in social programs as part of an integrated health-care effort, as well as treating minor conditions in the field or referring patients to non-ED health resources [24–27]. CPN has been proven to have favourable outcomes [25]. However, as the discipline is still evolving, consensus is lacking on what community paramedics (CPs) can contribute to the healthcare of elderly and within the wider healthcare system [25].

In light of this, CPN could potentially be further evolved to become an additional resource for RACFs through the provision of more specialised care, especially in palliative and end-of-life situations. The rapid expansion of paramedic practice has left the profession with developing professional and clinical boundaries, and current frameworks may no longer be consistent with actual practice or the expectations of healthcare consumers. Exploring new and emerging models of healthcare that align paramedicine with the changing landscape is essential to guide the advancement of this profession [28]. In this systematic review, we specifically focus on emerging models of healthcare for elderly patients. Our aim is to identify the best available evidence of the role CPN currently has in healthcare delivery for elderly patients and the impact that this might have on the wider healthcare system.

We addressed the following research questions:

1. What role does CPN currently have in healthcare delivery for elderly adults?
2. What is the impact that this healthcare delivery by CPs could have on patient health and the wider healthcare system?
3. Is there evidence to support CPN involvement in healthcare delivery in RACFs and PEOL care?

## Methods

### Data source and search strategy

A systematic review of the literature was conducted using the PRISMA reporting guidelines [29]. Because we aimed to uncover the international evidence for CPN, to confirm current practice, and identify and inform areas for future research, a systematic review was deemed more appropriate than a scoping review [30]. Due to funding related time constraints, we did not register a protocol with PROSPERO [31].

The aim was to identify evidence for the role that community paramedicine could have in the care delivery for elderly people. MEDLINE, Embase, CINAHL, and Web of Sciences were searched to identify all relevant full-text articles in English published until October 3, 2019. The search terms covered the following areas, using MeSH terms and text words: (1) emergency medical services, including ambulance and paramedic; (2) palliative and end-of-life care; (3) aged care, including nursing homes and health services for the aged; and (4) terms related to community paramedicine. The search strategy is available in a supplementary file (see Additional file 1). To ensure the widest possible search, no date filter was used. During the course of the search, references citied in systematic reviews were screened for potential further references.

### Inclusion and exclusion criteria

The inclusion and exclusion criteria are shown in Table 1. Studies that looked at paramedics in the traditional emergency role without further training were deemed not relevant as the differentiating factor for the CP role is working beyond the traditional scope with additional training. We included studies with elderly patient populations, palliative care patients, and RACF patients. In order to not exclude relevant studies, study designs could include randomised controlled trials, pre-post designs, cross-sectional, cohort, and qualitative studies.

**Table 1:** Inclusion and exclusion criteria

Criteria	Inclusion	Exclusion
Language	English language	Non-English
Article type	Peer-reviewed	Abstract Poster Editorial Letter Chapter
Type of studies	Intervention Studies Quantitative and qualitative studies Randomized control studies	Case Studies
Patient Problem	Elderly patients Palliative or end-of-life care patients Residential aged care facility residents	Studies of children, adolescents, young adults.
Intervention	Interventions focussing on paramedics with extended skills/community paramedics caring for elderly patients.	Interventions focussing on paramedics solely as transport role. Interventions focussing on community paramedicine for children. Intervention focusses on community paramedic training.
Control	N/A	N/A
Outcome	The role of community paramedicine (CP) in care delivery for elderly adults Impact of CP on health care system Impact of CP on patient health The role of CP in Residential Aged Care Facilities and Palliative and End-of-Life care	N/A

### Critical appraisal

The Joanna Briggs Institute (JBI) appraisal checklists for cross sectional, qualitative, cohort, and randomised controlled trial studies were used to assess the methodological quality of the articles [32].

The authors looked for strengths and weaknesses in each article by answering ‘Yes’, ‘No’, ‘Unclear’, or ‘Not Applicable’ to each question. For this quality assessment, a numerical value of one was attached to each ‘Yes’ answer, and the ‘Not Applicable’ answers were not included. The number of ‘Yes’ answers was then divided by the total number of applicable questions of the checklist. Studies with a score of 70% or more were considered to be at low risk of bias [33–35]. As the aim of this study is to inform practice with the best available evidence, 70% was chosen as the threshold for inclusion, which was agreed upon prior to the commencement of the critical appraisal, as recommended by the JBI reviewer’s manual [36].

### Data extraction and analysis

One author (JV) extracted the data in line with a piloted form looking at study characteristics and main findings. The first round of data extraction summarised the study characteristics, these included year, country, patient population, role or task of the paramedic, and the paramedic’s extra training. The second round focussed on study participants, aim of the study and the main findings.

JV undertook the first round of identifying emerging themes. Each paper was viewed through the prism of the role of CP and a process of narrative synthesis was used to summarise the current state of knowledge and understanding [37], identifying the role of CP in the studies and the impact they had on the health care system. A narrative synthesis approach has been used elsewhere in health services research [38], and is well suited to examining questions looking at effectiveness or cost effectiveness, appropriateness, or feasibility of implementation of interventions. It also suits a research question that dictates the inclusion of a wide range of research designs, producing qualitative and quantitative findings [37], which was deemed appropriate for this review as we did not exclude articles based on research methodology.

In an iterative process, two other authors (BT, ES) independently reviewed the data and adjusted the summarised themes by expanding or merging themes and subheadings. Finally, all authors reached an agreement on the main themes.

## Results

### Search outcomes

**Fig. 1:**
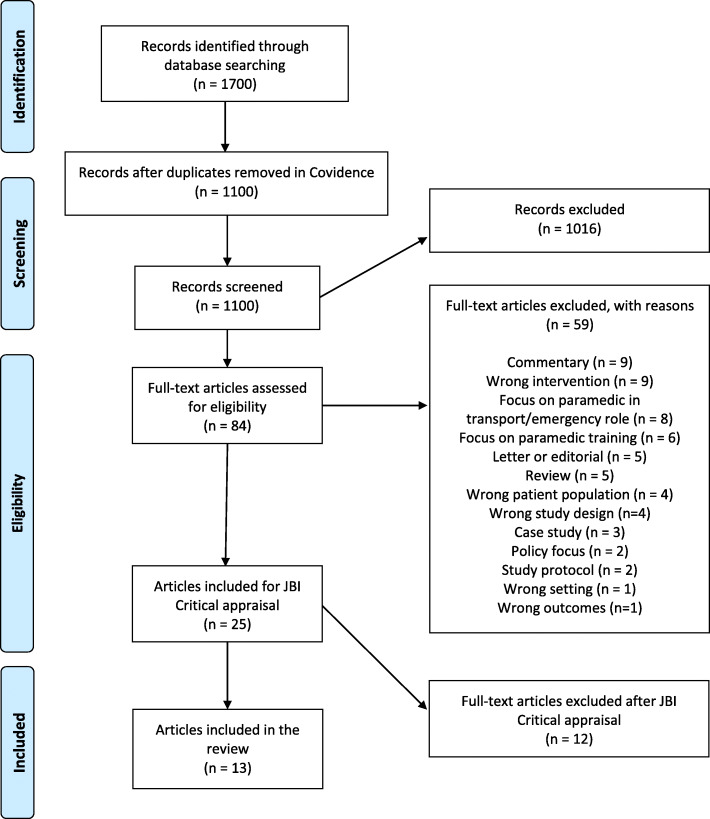
PRISMA Flow diagram

The literature search identified exactly 1700 records (Fig. 1). Citations were screened using Covidence [39], which is a web-based software platform that streamlines and supports the process of systematic reviews. Six hundred duplicates were identified. The title and abstract of the remaining 1100 papers were screened by two reviewers independently. All authors acted as reviewers at each stage of the screening process. Articles were excluded according to the criteria summarised in Table 1. For full-text screening, 84 papers were retrieved, which were read in detail by two reviewers independently and the exclusion and inclusion criteria were applied (Table 1). A further 59 were excluded, resulting in 25 studies being critically appraised. The quality assessment was undertaken independently by authors JV and BT, and no overall disagreement was found. Twelve articles did not meet the criteria and were excluded from the review [40] (see Additional file 2).

### Study characteristics

A total of ten studies, which were reported across 13 articles (Table 2), were included in the systematic review, [26, 41–52]. The majority of the studies were conducted in Canada (6), and the rest in the USA (3) and the UK (1). All of the studies focussed on a senior or elderly population. The participant sample size varied across the studies with the larger studies ranging between 1092 and 4081 participants, and the smaller qualitative studies between 21 and 94. The participants were a mix of elderly patients, family members, community members, and healthcare workers The studies used a mix of methods, with five qualitative studies, three cross sectional studies, three randomised controlled trials, and two cohort studies.

**Table 2:** Study, patient and role of paramedic characteristics

	Author(s) Year	Country	Study Design	Patient characteristics	Role of Paramedic	Paramedic’s additional training
1	Abrashkin et al. 2016 [41] Abraskhin et al. 2019 [42]	USA	Observational studies	Elderly patients, home-bound with two or more chronic condition, enrolled in the advanced illness management (AIM) program.	*Assessment* • In-home evaluation and treatment of acute illnesses. • Telephone triage with acuity rating code *Communication* • Discussion of emergency department (ED) transport or attempted home interventions with physician through video or telephone conferencing.	Additional 40 h of instruction in geriatrics and home-based primary care through didactic training and physician observation.
2	Agarwal et al. 2018 [43] Agarwal et al. 2019 [44]	Canada	Cluster randomized controlled trials	Residents aged 55 years and older of a subsidized apartment building.	*Assessment* • Assessment of cardiovascular, diabetes, and fall risk. *Education* • Disease prevention and health promotion sessions. *Referral* • Identification of high-risk patients and referral to healthcare • Targeted referral to community resources • Referral to urgent care or ED in case of emergency medical incident. *Communication* • Regular communication of participants’ health information with their family physician.	Community paramedics (CP) undertook online modules on chronic diseases, their risk factors, risk assessment using validated tools, and health promotion methods (approximately 4 h of training); webinars were used for CP@clinic database training (1 h of training); in-person observation using a train-the-trainer model was expected by each paramedic service for at least 1 clinic session of 2–3 h duration.
3	Bennett et al. 2018 [45]	USA	Pre/post-test with a comparison group study design.	Frequent users of the ED and have at least 1 chronic disease.	*Assessment* • Home safety assessments • General assessments: medication reconciliation, blood glucose, and weight checks • Cardiovascular and respiratory care • Post-discharge follow-up *Education* • Patient education *Referral* • Connecting participants to resources for primary care delivery • Applications for benefits • Referral to urgent care or ED in case of emergency medical incident.	200 h didactic training & 100 h local clinic time, with at least 10 years in Paramedic Services and at least 4 years at the local county Paramedic Services.
4	Brydges et al. 2015 [46]	Canada	Interpretivist qualitative approach	Seniors	*Referral* • Initiating referrals to community services	Varied forms of education: training by continuing medical education, email communication, or none at all.
5	Brydges et al. 2016 [26] Agarwal et al. 2017 [47]	Canada	Brydges: Interpretivist qualitative approach Agarwal: A prospective pre-post approach for intervention study	Residents aged 55 years and older living in a subsidized housing building	*Assessment* • Two four-hour sessions per week. • Cardiovascular, diabetes and fall risk assessment. *Education* • Providing education based on risk assessment, including information on local resources. *Referral* • Developed individualised action plan directing participants to available community resources. • Standard protocol was followed for emergency action. *Communication* • Participant’s information was faxed to family physician once a month.	Half-day long training session by public health nurse and family doctor in taking blood pressure, conducting health assessment, educating residents and using the program’s database.
6	Dainty et al. 2018 [48]	Canada	Qualitative study	Patients living with one of three major chronic diseases: diabetes mellitus, congestive heart failure, and chronic obstructive pulmonary disease.	*Assessment* • Scheduled home visits at 3-month intervals and follow-up and emergency home visits to assess and treat patients.	Six-week intensive course in chronic disease management.
7	Jensen et al. 2014 [49]	Canada	Qualitative approach	LTC residents	*Assessment* • Managing acute situations in the patient’s home environment. *Referral* • Arranging transfers to occur at a time where the receiving department can see the patient more quickly.	Two weeks of additional training in: (1) geriatric assessments and management; (2) EOL care; (3) primary wound closure techniques (suturing, tissue adhesive); and (4) point of care testing.
8	Kant et al. 2018 [50]	USA	Case series / Mixed methods	Geriatric patients	*Assessment* • Home-based episodic care	–
9	Mason et al. 2008 [51]	UK	Cluster-randomized controlled trial	Patients aged 60 years or older with minor injury or illness.	*Assessment* • Assess and, where possible, treat patients in the community. *Referral* • Referral to ED, general practitioner, district nurse, or community social services.	Three-week lecture-based program to assess and (where possible) treat older people in the community, followed by 45 of supervised practice.
10	O’Meara et al. 2015 [27]	Canada	Observational, ethnographic research	–	–	Enhanced knowledge and broader understanding of health issues.

### The role of the community paramedic

In regard to the care delivery for elderly adults, all studies, with the exception of one [52], described different aspects of the role of the CP (Table 2). The roles varied somewhat across the studies. Four main themes emerged: assessment, referral, education, and communication.

#### Assessment

Most studies reported on assessment as part of the role of the CP [26, 41–45, 47–50]. The evaluations were conducted in the patient’s home or one-on-one in the building where they lived. In some studies, the evaluation focussed on whether the patient should be transferred to ED or the treatment of acute illnesses could be completed at home [41, 42, 48–51]. Here, the aim was to prevent unnecessary ED transports and visits, and hospital admissions. Other studies reported a preventative risk assessment, aiming to identify high-risk patients and to provide education or referral where needed [26, 43–45, 47, 48]. The most common risks tested for were cardiovascular, diabetes, weight checks, and fall risks. Bennett et al. also included post-discharge follow-up assessments as well as home safety assessments as part of the role of the paramedic [45].

#### Referral

The next most commonly identified aspect of the paramedics’ role was referral. Based on the identification of high-risk patients, CPs would ensure targeted referral to community resources, community services, or to specific primary healthcare workers [26, 43–47, 49, 51]. Most studies that provided risk assessments, also provided referral to urgent care or ED in case of an emergency medical incident. One study reported that the use of CPs allowed for transfers at a time where the receiving department could see the patient more quickly [49].

#### Education

Within the studies focussing on preventative risk assessment, education was another important preventative tool [26, 43–45, 47]. Studies encompassing an education component included those focussing on general disease prevention and health information sessions [43, 44], as well as studies which provided specific education and information on local resources based on the patient’s level of risk [26, 47].

#### Communication

The last aspect of the role of a CP was communication. This was generally described as regular communication of the patient’s health information with their family physician [26, 43, 44, 47]. In one study, the CP communicated with a physician to discuss ED transport or in-home treatment [41, 42].

#### Additional training

The observation that the role of the paramedic varied across the studies might be related to the lack of additional training that were reported throughout the studies reviewed (Table 2). Training ranged anywhere from half a day [26, 47] to a six-week intensive course [48]. Training varied in duration as well as content. Abrashkin et al. stated that their additional training included 40 h of instruction in geriatrics and home-based primary care through didactic training and physician observation [41, 42]. Agarwal et al. included online modules on chronic diseases and risk factors combined with webinars and in-person observation [43, 44]. Jensen et al. concluded that “soft skills” and the ability to handle difficult conversations were essential for a CP and that current education and training was inadequate [49].

### The impact on care

Our second research question aimed to investigate how a CP working in care delivery for elderly patients might have impact on both the patient’s health and the wider healthcare system. The findings for each study can be found in Table 3 with overall findings and potential challenges discussed in detail below (Table 3).

**Table 3:** Impact of the community paramedic role in the care of the elderly on patient health and the wider healthcare system

	Author(s)	Study participants	Aims / Outcome Measures	Main findings/themes
1	Abrashkin 2016 [41] Abrashkin 2019 [42]	***Patients*** *2016* (*n*=1602) Median age: 83 years Had at least one emergency response (*n*=773) Used CP at least once (*n*=404) Used only traditional Paramedic Services (*n*=369) *2019* (*n*=1159) Average age: 86 years	• Emergency Department (ED) transport and hospitalisation rate after Community Paramedic (CP) visit. • Response time, time on scene and medications administered. • Post-visit feedback • Patient/caregiver satisfaction • Descriptive data	*Healthcare system* • In 78% of CP responses, individuals were evaluated, treated, and remained at home. • One or more treatments were administered in 27.6% of CP responses. • After transport to ED, hospital admission rates were significantly higher for individuals transported after a CP response than a traditional Paramedic Services’ response. • Only 1.7% of patient who received a CP visit and were not transported to ED, were subsequently seen in an ED within 24 h of response. • Transport rates were not significantly different for those with ACP or without. *Patient health* • Individuals seen by CP were older, have more activity of daily living (ADL) dependencies, and have a do-not-resuscitate order than those using only traditional Paramedic Services’ responses. • Both groups had high rates of advance care planning. • High acuity responses were the most common. *Patient satisfaction* • Patient/caregiver satisfaction was high, stating that they felt that the goals of care were accounted for and they would use CP in future medical emergency. *General* • CP program can provide a safe and effective option for responding to and treating frail older adults in their home, avoiding transport to ED and likely hospitalisation.
2	Agarwal 2018 [43] Agarwal 2019 [44]	***Patients*** *2018* Intervention (*n*=455) Control (*n*=637) *2019* Intervention (*n*=2009) Control (*n*=2072) Both groups: Mean age of 70 yrs	• Monthly ambulance calls at the building level per 100 apartment units. • Secondary health, knowledge, behaviour outcomes (individual level), including changes in risk factors. • Changes in blood pressure (BP), lifestyle risk-factor measures, health-related quality-of-life (HRQoL), and quality-adjusted-life-years (QALYs).	*Healthcare system* • There were significantly less calls in the intervention buildings. *Patient health* • The intervention improved risk factor profiles and the health-related quality of life of the participants. • Three HRQoL domains (self-care, usual activities, pain, and discomfort) and overall QALY significantly improved among the intervention group. • The intervention participants had improved CANRISK, implying the intervention had an impact in reducing the participants’ risk of developing diabetes. • The intervention participants’ blood pressures showed a significant and sustained decrease.
3	Bennett 2018 [45]	***Patients*** Intervention (*n*=68) Average age 57.6 yrs. Control (*n*=125) Average age 55.4 yrs	• Satisfaction of participants • Screening and compliance rates • Appropriate use of care • (Non-emergent) calls to Paramedic Services • Time spent on scene • Time to return to services • ED visits • Inpatient admissions • 30-day readmission rate • Costs	*Healthcare system* • The program was cost-effective. • Within healthcare utilisation, care was moved from ED and inpatient to outpatient and medical home-based: significantly less transports and less ED visits. *Patient health* • Patients experienced an improved level of care and improved outcomes. *Patient satisfaction* • The patients were satisfied with the service.
4	Brydges 2015 [46]	***Paramedics*** (*n*=23)	Get a better understanding of the ways in which paramedics experience and participate in community-based referral programs	*Integration* • The referral program confronted the paramedic with an alternative approach to patient care which is in conflict with the traditional values and beliefs grounded in emergency response. • Participants felt that they had an inadequate knowledge base on the referral programs, due to inadequate education. • Feedback from the services following a referral, provided to the paramedics, was seen as inadequate. • Participants felt that they, the employer and sometimes even the CCAC were not held accountable by the program. • Participants saw the referral program as a way that they could step into the role of patient advocates.
5	Brydges 2016 [26] Agarwal 2017 [47]	***Patients*** *2016* (*n*=15) Age 63–89 *2017* (*n*=79) Mean age 72.2 yrs	Changes in: • Number of emergency Paramedic Services calls from the seniors’ residence building • Mean blood pressure of participants • Diabetes risk profile To understand the patients’ experience with the community paramedicine program.	*Healthcare system* • The number of emergency calls dropped by 7.1% after 6 months, and by 25% after 12 months. • The program could decrease healthcare costs. *Patient health* • Prevalent risk factors assessed by the CP were: high waist circumference and elevated body mass index, and stress. • Systolic and diastolic BP dropped by the 3rd and 5th CP visit respectively. • 15% of patients dropped in risk category for diabetes after 6 to 12 months. *Patient satisfaction* • The paramedics were seen as caring, respectful and trustful healthcare providers. The sessions were an individualised experience. • Paramedics had a dual role as both health advocates and as emergency experts. • Patient felt that it was reassuring knowing someone was taking care of them.
6	Dainty 2018 [48]	***Patients*** (*n*=30) Age 42–95 ***Family members*** (*n*=10)	To understand the experiences and perspectives of patients and families involved with the Expanding Paramedicine in the Community (EPIC)	*Patient health* • Patients indicated they learned new health information in management of their disease. *Patient satisfaction* • Participants developed a unique relationship with the EPIC paramedics, which they saw as important, trusted, and essential members of their healthcare. • Paramedics were seen as exceeding their expectations and went ‘beyond the call of duty’ to take care of the patients. • EPIC recognised the patients’ and provided a safety net in times of exacerbation and was a source of health education and accountability.
7	Jensen 2014 [49]	***Healthcare workers*** (*n*=21)	To better understand the experience of those directly and professionally involved in various aspects of the extended care paramedics (ECP).	*Training* • The ECPs current training was seen as inadequate. • “Soft skills” and the ability to handle difficult conversations were seen as an essential for an ECP. • ECPs saw decision making as a significant element of their role and reported longer time on the call as they discuss the situation with staff and/or family. *Integration* • The ECPs are able to bridge communication with the physician or family, and effective communication was seen as important to build relationships with LTC staff. *End-of-Life (EOL)* • There was a mixed view on how frequent people thought ECPs are involved in EOL care. • Multiple participants saw the ECP role as permitting enough flexibility to pause and determine what the best option is for an EOL patient. • Advanced care directives were seen as useful, but they can also be confusing.
8	Kant 2018 [50]	***Patients*** (*n*=35) Mean age 87.8 ***Geriatric primary care team*** (*n*=10)	To understand the use and potential impact of a community paramedicine program on patient care, including understanding provider perspectives.	*Healthcare system* • The majority of patients could be treated at home and did not require higher levels of care • Potential reduction of unnecessary ED visits/hospitalisations, decreasing the risk of hospital acquired complications. *Patient health* • The program was seen as beneficial as it gave patients greater access to care and provided reassurance and relief for caregivers. *Integration* • Health workers felt that they were notified too late about their patients regarding acute issues or general documentation. But the CP visit notes did give them a better insight into the patients’ home and social situation. • Geriatric team members expressed concern regarding the ability of CPs to provide appropriate geriatric-focussed care. They also questioned the integration of the program with primary care and whether the patients with acute needs should contact the service or their primary care provider first. They did see the role of CP as first responder more clearly in on-call situations. • The program is unique in that it uses a nurse practitioner or physician assistant as well as a CP.
9	Mason 2008 [51]	***Patients*** Intervention (*n*=1118) Control (*n*=907)	To evaluate the safety of the clinical decisions made and appropriateness of care provided by the Paramedic Practitioners working within the new service. This was measured by unplanned ED attendance within 7 days of paramedic visit.	*Healthcare system* • There was an overall 10.8% return visit rate at the ED within 7 days. • There were more returns visits in the intervention group, but the proportion returning with a related condition in both groups was not found significantly different. *Patient health* • There was also no difference in mortality at 28 days. • The paramedics in this trial are assessing and treating older people in a manner that is as safe as the standard care provided by Paramedic Services and ED. • Suboptimal care was judged by either or both ED clinician in 2.1% of the patients not admitted after their index episodes, but this was not significantly different between the groups.
10	O’Meara 2015 [27]	A range of participants: • paramedic service managers • paramedics • educators • physicians • nurses • other health professionals • patients • community members	Identify and describe the nature of the relationship between public engagement and the integration of CP with local health, aged care, and social services.	*Engagement* • The program used a range of engagement strategies to plan and implement key elements of their program. • The program developed elements of inclusiveness thorough community paramedic interactions with clients, families and carers in public and home settings. *Integration* • Participants recognised the important role of the program in integrating different services for patients and helping them navigate through the health, aged care and social service systems. • It was not always obvious to some participants how the CP program is integrated with the local health system. • It was noted that the longer-term sustainability of the CP program is reliant on strong integration with existing services. • The program played a variety of roles.

#### The patient’s health

Several studies reported improved patient health outcomes [26, 43–48, 50]. Prevalent risk factors assessed by the CPs were weight, cardiovascular, diabetes, and fall risks. One study reported that 15% of patients experienced a drop in risk category for their diabetes after 6 to 12 months [26, 47], and similar results were seen in the study completed by Agarwal et al. [43, 44]. Mason et al. reported no difference in mortality between patients seen by CPs or Paramedic Services [51]. Additionally, two studies reported reduced patient blood pressures in association with a CPN program [26, 43, 44, 47]. Patients in these studies experienced improved levels of care and personal health [26, 43–45, 47], learned new health information to manage their disease [48], and the CPN programs gave the patients greater access to care, and provided reassurance and relief for caregivers [50].

#### Patient satisfaction

Patient satisfaction was reported as being high [26, 41, 42, 45, 47, 48]. Patients felt that the goals of care were accounted for and they would use the CPs in future medical emergencies [41, 42]. The paramedics were seen as caring, respectful and trustworthy healthcare providers [26, 47, 48]. Patients felt it was reassuring to know someone was taking care of them [26, 47]; the paramedics were seen as going ‘beyond the call of duty’ to take care of patients [48].

#### The wider healthcare system

The majority of studies saw a reduction in emergency calls, transport to ED, ED visits, or hospitalisation [26, 41–45, 47, 50]. Abrashkin et al. reported that in 78% of the CPs’ responses, the individuals were evaluated, treated, and remained at home [41, 42]. They found that if the patient did have to be transported, hospital admission rates were significantly higher for individuals transported after a CP response than a traditional Paramedic Services’ response, indicating the CPs ability to identify the sickest individuals who need and want inpatient treatment. Another study reported that the number of emergency calls dropped by 25% after 12 months [26, 47]. Mason et al. investigated the safety of the clinical decisions made by the CPs through unplanned ED attendance within 7 days of the visit. They concluded that the CPs provided care at least as safe as the standard care provided by the Paramedic Services and the ED. The programs were also seen as a potential way to decrease healthcare costs [26, 45, 47]. The CPN programs were seen as a safe and effective option for responding and treating older adults at home [41, 42] and reducing the strain on Paramedic Services and ED by moving care from ED and inpatient to outpatient and medical home-based care [45].

#### Integration

As CPN is a new shift in the way this workforce is used, integration was seen as a challenge in some studies [46, 50, 52]. The referral program evaluated by Brydges et al. confronted the paramedic with an alternative approach to patient care which is in conflict with the traditional values and beliefs grounded in emergency response [46]. Another study described this confusion in the traditional emergency role versus a more primary care role in terms of where the CPN program would sit [50]. O’Meara et al. noted that their participating healthcare workers were not always clear on how the CPN program was integrated in the local health system. They did however recognise that the CPN program could play a key role in integrating different services and in helping patients navigate through the healthcare system [52]. They further noted that the CPN program currently plays a variety of roles within the healthcare system and that the long-term sustainability of the program is reliant on strong integration with existing services [52].

Communication between the CPs and other services could be improved according to some studies [46, 50]. It was, however, noted that the information provided by the CPN program was useful in providing a better insight into the patient’s home and social situation [50]. Jensen et al. found that the CPs were able to bridge the communication gap between the physician and family, and effective communication was seen as important to the building of relationships with Long Term Care (LTC) staff [49]. The CPs in this study collaborated with the physician and LTC staff in decision making and in the follow-up care [49].

### Residential aged-care facilities and palliative and end-of-life care

Our systematic review found limited high-quality evidence to support CPN involvement in the care delivery in RACFs and PEOL care specifically, although there was high quality evidence for the role of CPN in care of elderly patients. Jensen et al. reported on a paramedic involvement in EOL in a long-term care facility [49]. The paramedics in this study managed acute situations in the patient’s home environment and arranged transfers where necessary. They received extra training in geriatric assessment as well as EOL care. The other healthcare workers involved thought that the paramedics’ current education and training was inadequate, but they were good in bridging communication between the different parties involved. The participants in this study had mixed views on how frequently CPs were involved in EOL care. They did, however, see the role as offering flexibility to pause and determine the best options for the EOL patient. Advanced care directives were seen as useful in terms of guiding the care of the patient, although some participants emphasised that they could be confusing as the wording used was not always clear to the CPs.

Two other studies were conducted in senior subsidized housing buildings with residents aged 55 and over [26, 43, 44, 47]. The role of the community paramedic was preventative and entailed weekly drop-in sessions assessing and promoting health. Both studies reported positive impact on patient health and the wider health system. Although these studies did not take place within an RACF, a similar model might work within an RACF.

## Discussion

### Main findings

In this systematic review we aimed to identify evidence of the CP role in care delivery for elderly patients, with an additional focus on palliative care, and the impact that this new role might have on the wider healthcare system. All studies included fulfilled the JBI critical appraisal criteria, with most scoring high on the criteria. In terms of care delivery for elderly adults, the role of a CP was often a combination of four aspects: assessment, referral, education and communication. Additional training in the studies varied considerably, and there was no evidence of the international curriculum for CPN being used [53]. The studies provided clear evidence that the programs had a positive impact on the health of the patient, had high patient satisfaction, and could reduce the stress on other parts of the healthcare system. How CPN programs are integrated and varied from the traditional emergency role of a paramedic was seen as a challenge. Good integration is important to ensure long-term sustainability. Limited evidence was available on CPN involvement in care delivery in RACFs and PEOL care but the evidence to utilise CPs in the care delivery for elderly adults is promising.

### Interpretation of findings

#### Role of paramedic

The role of the CP varied across the studies, which is not surprising given that the concept is still evolving. This might also be linked to the finding that the training varied across the studies, and that the studies showed no evidence of using the international curriculum as a guide to additional training for CPN [53]. An additional explanation could be that the studies focussed on different areas of care, and therefore the training was aligned with the healthcare area. The four identified aspects (assessment, referral, education and communication) all seem important in delivery of care for elderly adults. To ensure consistency and safe care delivery and longer-term sustainability, adherence to the international curriculum is important. Simpson et al. found that paramedics experience confusion over their role and that this has a substantial impact on the decision-making process when caring for older fallers [54]. In their study of fallers, they concluded that education and training play a part in the decision-making as well as the paramedic’s attitude toward patients [54]. Thus, more uniformity in training and improved role clarity could have a positive impact on providing patient-centred, good quality care to the elderly population.

Although the issue of how education and training might impact on outcomes was not one of our primary aims, it would be of interest if the identified programs were to be more widely implemented. Most of the included studies were from the US or Canada, which explains that the entry-to-practice education on death and dying for paramedics was lacking, as well as the reliance on short courses to upskill participants. In contrast, in Australia and the UK paramedics are educated in universities alongside other health professionals, with all health professional programs sharing content. In Australia it has been identified that paramedic education in the area of death and dying (including palliative care) could be enhanced [55].

Paramedicine as a profession has a unique position in the community and healthcare system. Paramedics have high autonomy in their work and they have a role in establishing or maintaining the trust from the community [28]. These characteristics support a shift beyond an exclusively emergency role. Such a shift, however, requires the profession to re-think how it is defined, which might cause tension with the more traditional views of paramedic practice. Until the new potential roles are fully understood and tested, these tensions may persist [28]. The CSA Group in Canada has developed a clear definition of a CP:

“A community paramedic is defined as a paramedic who has completed a formal and recognized educational program and has demonstrated competence in the provision of health education, clinical assessment and monitoring, point-of-care diagnostics, and treatment modalities within or beyond the role of traditional emergency care and transport.” (p.13) [56]

They add to this that the goal of any CPN program should be to “promote the patient’s access to the right care, delivered by the right provider, at the right time, resulting in the best outcomes and the most effective and efficient use of resources. The foundation of any program will be dependent on stable and sustainable partnerships among numerous community-based agencies, teams and organizations.” (p.10) [57]. Therefore, it is recommended to adhere to the above definition and goals to ensure consistency in CPN programs.

#### Reconfiguring healthcare workforce

As mentioned earlier, there is a universal shift in the way the healthcare workforce is utilised, due to shortfalls in workforce supply or distribution. The workforce has diversified with for example specialised roles for nurses and other health professionals, of which CPN is an example. De Bont et al. categorised the development of new extended roles into two groups: (i) specialised roles which occupy a narrowly defined area of expertise, (ii) generic roles, which have a broader scope and cover a larger part of the care pathway. They concluded that the extended roles develop new ways of health service provision that suit healthcare organisations, encouraging and shaping organisation-oriented healthcare delivery [1].

A challenge to the integration of CPN programs is that the extended scope of practice may be classified as both a specialised and a generic role. As the CPs, especially in the care delivery for elderly adults, receive specific additional geriatric, risk assessment, or PEOL training, the role has developed according to the increasing complex needs of the elderly population. However, the CP also takes on a role of health advocate, educator, and point of reference between community health services, physicians, and hospitals, covering a larger part of the care pathway. The profession continues to be seen by other healthcare professionals as being exclusively as an emergency health profession. Moreover, good integration within the healthcare system is key to ensuring the long-term sustainability of any CPN program.

#### RACF and PEOL care opportunities

Our review found limited evidence for the involvement of CPs in the care delivery in RACFs and PEOL care. However, two studies were conducted in subsidized building for seniors, where the CPs had weekly sessions during which they checked for health risks and provided health promotion and education to residents [26, 43, 44, 47]. This setting is similar to a setting in an RACF, suggesting that a similar model might work in RACFs. As RACFs residents are an especially complex and vulnerable population, with high levels of frailty, functional impairment, and comorbidities including cognitive impairment, regular preventative and check-in health sessions might improve the health of these patients and provide access to quick emergency transport if necessary. One study in our review found that the CPs were effective in bridging the communication gap between the patients, family and physicians, as well as the RACF staff. They could potentially play a central role in improving and maintaining the health of residents in RACFs.

In PEOL care, many different healthcare professionals are involved, resulting sometimes in fragmented care. Another issue is the large discrepancy in numbers between those who wish to die at home and those that actually do so. A recent report on the sustainability of community PEOL care services, found that workforce shortages, along with pain medication and symptom management are key aspects that need to be addressed if patients’ wishes in regard to place of care and place of death are to be addressed [58]. CPN could potentially play a role in symptom management and fill workforce gaps, whilst being a central communicator to ensure less fragmented care. The one study in our review that addressed EOL, found that the CPs had inadequate education and training, even though they did have the flexibility and time to make correct decisions for the patients [49].

The role CPN could have in RACFs and PEOL warrants further research, as the role could be a promising initiative to improve the health and specialised care for the older community.

### Implication for research and practice

This review provides evidence to show that CPN involvement in the care delivery of elderly adults is increasing with positive results both for the population and the healthcare system. Based on the limited evidence available, this review has found that CPs could potentially provide the specialised care needed in these areas, whilst simultaneously reducing the pressure on hospitals and other healthcare providers.

### Limitations and strengths

Since CPN is a developing concept, there is limited high-quality evidence available. Out of the potential 25 studies, only 13 satisfied the JBI critical appraisal criteria. However, the majority of the 13 studies included in this review scored highly on the JBI critical appraisal benchmark.

Another limitation of this review is that it could be argued that it is challenging to compare outcomes, due the variation in CP training across the studies. Nevertheless, the quality of each study was high, and the outcomes were consistently positive. This, in turn, facilitated the identification of overarching themes.

We see the results of the different studies as a strength. The shift to utilising CPs is promising on a number of levels, from patient health to the wider healthcare system. Our review is one of the few to summarise the evidence available in the evolving practice of CPs.

## Conclusions

The healthcare system is often overloaded and ever changing. With workforce issues, rather than focussing on more healthcare workers, there is a growing international trend to develop new models of healthcare delivery to address the demand. With an ageing population, the demand on care delivery for elderly adults is inevitably increasing. The use of CPs in the care delivery could be beneficial to both patients’ health and the wider healthcare system. CPs already play a promising role in improving the care of our elderly population. With consistent adherence to the training curriculum and effective integration within the wider healthcare system, CPs have the potential to take on specialised roles in RACFs and PEOL care.

## Supplementary Information

**Additional file 1.** Database Search Strategy – Overview of applied search strategy across the databases.

**Additional file 2.** JBI Critical appraisal – Overview of critical appraisal results of the 25 studies.

## Data Availability

All data generated or analysed during this study are included in this published article and its supplementary information files.

## Abbreviations

CP: Community Paramedic
CPN: Community paramedicine
PC: Palliative care
EOL: End-of-life
PEOL: Palliative and end-of-life care
RACF: Residential aged care facility
ED: Emergency department
JBI: Joanna Briggs Institute
