# Supplementary material for: Reshaping healthcare delivery for elderly patients: the role of community paramedicine; a systematic review
Source: BMC Health Serv Res. 2021 Jan 6;21:29. doi: 10.1186/s12913-020-06037-0 (PMC7789625; doi:10.1186/s12913-020-06037-0)
Supplement: Supplementary file 1 — Additional file 1. Database Search Strategy – Overview of applied search strategy across the databases. [file 12913_2020_6037_MOESM1_ESM.docx]

**Additional file 1.** Database Search Strategy

**MEDLINE**

1. Allied health personnel/ OR Emergency medical technicians/ OR Emergency Medical services/
2. (“emergency medical” ADJ (technician* OR service* OR practitioner*)).mp.
3. (Paramedic* OR EMT OR EMS).mp.
4. Ambulances/ OR ambulance*.mp.
5. 1 OR 2 OR 3 OR 4
6. Palliative care/ OR Terminal care/
7. ((terminal* OR palliative* OR “end of life”) ADJ1 (care* OR medicine* OR patient*)).mp.
8. 6 OR 7
9. Homes for the aged/ OR Nursing homes/ OR Health services for the aged/ OR Assisted living facilities/
10. “nursing home*” OR “aged care*” OR “gerontologic* care*” OR (residential* ADJ1 (care* OR home* OR facilit*)) OR “long term care*” OR “assisted living” OR “assisted care*” OR (retirement ADJ1 (facilit* OR home*)).mp.
11. 9 OR 10
12. 8 OR 11
13. 5 AND 12
14. (“mobile integrated” OR “community paramedic*”).mp.
15. 13 OR 14
16. Communit*.mp.
17. 13 AND 16
18. 15 OR 17
19. Limit 18 to English Language

**EMBASE**

1. Paramedical personnel/ OR Rescue personnel/ OR Emergency health service/ OR Ambulance/
2. (“emergency medical” ADJ (technician* OR service* OR practitioner*)).mp.
3. (Paramedic* OR Ambulance* OR EMS OR EMT).mp.
4. 1 OR 2 OR 3
5. Palliative therapy/ OR Terminal care/
6. ((terminal* OR palliative OR “end of life”) ADJ1 (care* or medicine OR patient*)).mp.
7. 5 OR 6
8. Homes for the aged/ OR Nursing home/ OR Elderly care/ OR Assisted living facility/
9. ( “nursing home*” OR “aged care*” OR “gerontologic* care*” OR “long term care*” OR “assisted living” OR “assisted care*”).mp.
10. (residential ADJ1 (care OR home* OR facilit*)).mp.
11. (retirement ADJ1 (facilit* OR home*)).mp.
12. 8 OR 9 OR 10 OR 11
13. (“mobile integrated” OR “Community paramedic*”).mp.
14. 7 OR 12
15. 4 AND 14
16. 13 OR 15
17. Community/ OR Communit*.mp.
18. 15 AND 17
19. 13 OR 18
20. Limit 19 to English Language

**Cinahl**

1. (MH “Allied Health Professions") OR (MH "Allied Health Personnel") OR (MH "Prehospital Care") OR (MH "Emergency Medical Technicians") OR (MH “Emergency Medical Services”) OR (MH “Ambulances”)
2. (emergency medical N1 (technician* OR service* OR practitioner*))
3. EMT OR EMS OR Paramedic* OR Ambulance*
4. 1 OR 2 OR 3
5. (MH “Palliative Care”) OR (MH “Terminal Care”)
6. (terminal* OR palliative OR “end of life”) N1 (care* OR medicine or patient*))
7. 5 OR 6
8. (MH “Nursing Homes”) OR (MH “Health Services for the Aged”) OR (MH “Gerontologic care”) OR (MH “Assisted Living”)
9. “homes for the aged” OR “nursing home*” OR “aged care*” OR “Gerontologic* care*” OR “long term care*” OR “assisted living” OR “assisted care*”
10. (residential N1 (care OR home* OR facilit*))
11. (retirement N1 (facilit* OR home*))
12. 8 OR 9 OR 10 OR 11
13. “mobile integrated” OR “community paramedic*”
14. 7 OR 12
15. 4 AND 14
16. 13 OR 15
17. “Communit*” OR (MH “Communities”)
18. 15 AND 17
19. 13 OR 19
20. 13 OR 19 narrow by Language: - English

**Web of Science**

1. TS=(“allied health personnel” OR “emergency medical technician*” OR “emergency medical service*” OR paramedic OR EMS OR EMT OR ambulance*)
2. TS=((palliative OR terminal OR “end of life”) NEAR/1 (care* OR medicine OR patient*))
3. TS=(“homes for the aged” OR “nursing home*” OR “aged care*” OR “gerontologic care*” OR “assisted living” OR “retirement home*” OR “retirement facilit*”)
4. 2 OR 3
5. 4 AND 1
6. TS=(“mobile integrated” OR “community paramedic*”)
7. 5 OR 6
8. TS=communit*
9. 5 AND 8
10. 6 OR 9
11. 6 OR 9 REFINED BY: LANGUAGES: (ENGLISH)
