# Supplementary material for: Reshaping healthcare delivery for elderly patients: the role of community paramedicine; a systematic review
Source: BMC Health Serv Res. 2021 Jan 6;21:29. doi: 10.1186/s12913-020-06037-0 (PMC7789625; doi:10.1186/s12913-020-06037-0)
Supplement: Supplementary file 2 — Additional file 2. JBI Critical appraisal – Overview of critical appraisal results of the 25 studies. [file 12913_2020_6037_MOESM2_ESM.docx]

**Additional file 2.** JBI Critical appraisal

Y = Yes

N = No

U = Unclear

NA = Not applicable

**Cross Sectional Studies**

| Study | 1 | 2 | 3 | 4 | 5 | 6 | 7 | 8 | Result % | Result |
| --- | --- | --- | --- | --- | --- | --- | --- | --- | --- | --- |
| Abrashkin 2016 | Y | Y | Y | Y | N | N | Y | Y | 75% | Incl. |
| Abrashkin 2019 | Y | Y | Y | Y | N | N | Y | Y | 75% | Incl. |
| Agarwal 2017 | Y | Y | Y | Y | Y | U | Y | Y | 88% | Incl. |
| **Carter 2019** | N | Y | Y | Y | N | N | Y | Y | 63% | **Excl** |
| **Chellappa 2018** | Y | N | U | Y | U | U | Y | U | 38% | **Excl.** |
| **Gerson 1992** | Y | Y | Y | U | U | U | Y | U | 50% | **Excl.** |
| Kant 2018 | Y | Y | Y | Y | Y | U | U | Y | 75% | Incl. |
| **Montgomery 2017** | N | N | U | U | N | N | U | U | 0% | **Excl.** |
| **Patterson 2016** | Y | Y | U | Y | U | U | U | Y | 50% | **Excl.** |

**Qualitative studies**

| Study | 1 | 2 | 3 | 4 | 5 | 6 | 7 | 8 | 9 | 10 | Result % | Result |
| --- | --- | --- | --- | --- | --- | --- | --- | --- | --- | --- | --- | --- |
| Brydges 2015 | Y | Y | Y | Y | Y | Y | Y | Y | Y | Y | 100% | Incl |
| Brydges 2016 | Y | Y | Y | Y | Y | Y | Y | Y | Y | Y | 100% | Incl |
| Dainty 2018 | Y | Y | Y | Y | Y | Y | N | Y | Y | Y | 90% | Incl |
| Jensen 2014 | N | Y | Y | Y | Y | Y | Y | N | Y | Y | 80% | Incl. |
| O’Meara 2015 | U | Y | Y | Y | Y | U | U | Y | Y | Y | 70% | Incl. |
| **Swetenham 2014** | N | Y | Y | U | U | N | N | U | Y | Y | 40% | **Excl.** |

**Cohort Studies**

| Study | 1 | 2 | 3 | 4 | 5 | 6 | 7 | 8 | 9 | 10 | 11 | Result % | Result |
| --- | --- | --- | --- | --- | --- | --- | --- | --- | --- | --- | --- | --- | --- |
| Bennett 2017 | N | Y | Y | Y | Y | N | Y | Y | Y | NA | U | 70% | Incl |
| **Jensen 2016** | N | U | Y | Y | N | Y | Y | Y | U | U | Y | 55% | **Excl** |
| **Siddle 2018** | U | Y | Y | Y | N | U | Y | Y | U | U | Y | 55% | **Excl.** |
| **Swain 2012** | U | Y | Y | N | U | Y | Y | Y | U | U | Y | 55% | **Excl.** |

**Randomised Controlled Trial**

| Study | 1 | 2 | 3 | 4 | 5 | 6 | 7 | 8 | 9 | 10 | 11 | 12 | 13 | Result % | Result |
| --- | --- | --- | --- | --- | --- | --- | --- | --- | --- | --- | --- | --- | --- | --- | --- |
| Agarwal 2018 | Y | U | Y | Y | NA | U | Y | Y | Y | Y | Y | Y | Y | 83% | Incl. |
| Agarwal 2019 | Y | Y | Y | NA | NA | NA | Y | Y | Y | Y | Y | Y | Y | 100% | Incl. |
| **Ashton 2017** | U | N | U | U | U | U | U | N | Y | Y | Y | U | U | 23% | **Excl.** |
| Mason 2008 | Y | NA | Y | NA | NA | NA | Y | Y | Y | Y | N | Y | Y | 88% | Incl. |
| **Shah 2018** | U | Y | U | NA | NA | Y | Y | U | Y | U | Y | U | U | 55% | **Excl.** |

**Prevalence Studies**

| Study | 1 | 2 | 3 | 4 | 5 | 6 | 7 | 8 | 9 | Result % | Result |
| --- | --- | --- | --- | --- | --- | --- | --- | --- | --- | --- | --- |
| **Gray 2008** | Y | Y | Y | U | Y | Y | Y | U | U | 66% | **Excl.** |
